# RAGE Differentially Altered *in vitro* Responses in Vascular Smooth Muscle Cells and Adventitial Fibroblasts in Diabetes-Induced Vascular Calcification

**DOI:** 10.3389/fphys.2021.676727

**Published:** 2021-06-07

**Authors:** Amber M. Kennon, James A. Stewart

**Affiliations:** Department of BioMolecular Sciences, School of Pharmacy, University of Mississippi, Mississippi, MS, United States

**Keywords:** AGEs, RAGE, vascular smooth muscle cells, adventitial fibroblasts, diabetes mellitus, vascular calcification

## Abstract

The Advanced Glycation End-Products (AGE)/Receptor for AGEs (RAGE) signaling pathway exacerbates diabetes-mediated vascular calcification (VC) in vascular smooth muscle cells (VSMCs). Other cell types are involved in VC, such as adventitial fibroblasts (AFBs). We hope to elucidate some of the mechanisms responsible for differential signaling in diabetes-mediated VC with this work. This work utilizes RAGE knockout animals and *in vitro* calcification to measure calcification and protein responses. Our calcification data revealed that VSMCs calcification was AGE/RAGE dependent, yet AFBs calcification was not an AGE-mediated RAGE response. Protein expression data showed VSMCs lost their phenotype marker, α-smooth muscle actin, and had a higher RAGE expression over non-diabetics. RAGE knockout (RKO) VSMCs did not show changes in phenotype markers. P38 MAPK, a downstream RAGE-associated signaling molecule, had significantly increased activation with calcification in both diabetic and diabetic RKO VSMCs. AFBs showed a loss in myofibroblast marker, α-SMA, due to calcification treatment. RAGE expression decreased in calcified diabetic AFBs, and P38 MAPK activation significantly increased in diabetic and diabetic RKO AFBs. These findings point to potentially an alternate receptor mediating the calcification response in the absence of RAGE. Overall, VSMCs and AFBs respond differently to calcification and the application of AGEs.

## Introduction

Approximately 34.2 million Americans live with type 2 diabetes mellitus (T2DM) (Centers for Disease Control and Prvention, 2020). T2DM hallmarks are high blood glucose or hyperglycemia, insulin dysfunction, and hyperlipidemia (Hameed et al., 2015; Zheng et al., 2018). Hyperglycemia is responsible for cellular metabolic changes resulting in tissue damage to organs, such as the kidney and heart (Brownlee, 2005; Hameed et al., 2015; Zheng et al., 2018; Oguntibeju, 2019). Additionally, diabetic patients exhibit mineral imbalances, such as hyperphosphatemia and hypercalcemia (Yamaguchi et al., 2011; Wang and Wei, 2017). Consequently, placing these patients at a higher risk for cardiovascular disease and stroke (Chonchol et al., 2009; McGurnaghan et al., 2019). Vascular calcification (VC) is a diabetic cardiovascular complication linked to hyperglycemia, hypercalcemia, and hyperphosphatemia (Giachelli, 2004, 2005; Demer and Tintut, 2008; Lau et al., 2010; Paloian and Giachelli, 2014; Mendes et al., 2017; Stabley and Towler, 2017; Wang et al., 2019). VC is the hardening of the medial layer of the macrovascular arteries, which contains a large population of vascular smooth muscle cells (VSMCs) responsible for dilating and constricting the arterial walls under physiological conditions. VC resulted from the deposition of hydroxyapatite minerals and was characterized by a phenotypic switch of VSMCs to osteoblast-like cells (Steitz et al., 2001; Rzucidlo et al., 2007; Chang et al., 2008). This change in phenotype and deposition of mineral leads to a stiffer vessel unable to operate normally, leading to an increased risk for a cardiovascular event (Chen et al., 2020). Also, hyperphosphatemia increases VC through increased action of the sodium phosphate co-transporter located on the surface of VSMCs (Lau et al., 2010; Crouthamel et al., 2013). The imbalance of phosphate inside the cytoplasm induces the cell to secrete hydroxyapatite crystals, promoting the osteogenic phenotype switch (Giachelli et al., 2001; Lau et al., 2010; Crouthamel et al., 2013). Hyperglycemia accelerates VC by activating the protein kinase C pathway and bone protein expression, osteopontin (Takemoto et al., 1999; Mori et al., 2002). When hyperglycemia and hyperphosphatemia are combined, VC was accelerated as demonstrated by Wang et al. (2019).

Hyperglycemia can also lead to an increase in advanced glycation end-products (AGEs) through interactions with amino groups on long-lived proteins to form non-enzymatic cross-links by the Maillard reaction (Singh et al., 2001). AGEs accumulate and bind to RAGEs (receptor or AGEs) to accelerate T2DM-mediated VC (Ren et al., 2009; Takeuchi and Yamagishi, 2009; Tanikawa et al., 2009; Yamagishi et al., 2012; Wei et al., 2013). Increased concentrations of extracellular AGEs will increase RAGE activation leading to downstream signaling through signaling proteins, such as extracellular-related kinase 1/2 (ERK 1/2), nuclear factor-κB (NF-κB), and p38 mitogen-activated protein kinase (p38 MAPK) (Tanikawa et al., 2009; Simard et al., 2015). These signaling molecules can activate several different stress pathways in VSMCs responsible for promoting an osteogenic phenotype switch, which is marked by the loss of the VSMC phenotype marker, α-SMA (α-Smooth Muscle Actin) as well as a change expression of osteogenic phenotype markers, such as osteopontin (OPN), osteocalcin (OCN), alkaline phosphatase (ALP), Msx2, and RunX2 or core-binding factor α1 (CBFα1) (Lian and Stein, 2003; Shao et al., 2005; Suga et al., 2011; Tada et al., 2013; Lok and Lyle, 2019). Within the vessel, another cell type, adventitial fibroblast (AFBs), has been relatively understudied. AFBs reside in the vessel’s outermost layer, where they cohabitate with vascular progenitor cells, pericytes, and immune cells (Kuwabara and Tallquist, 2017). Normally, AFBs are responsible for extracellular matrix deposition and secretion of cytokines and chemokines; however, AFBs can differentiate to “activated fibroblasts” under stressful conditions myofibroblasts, where they begin expressing α-SMA and increase migration toward sites of injury (Baum and Duffy, 2011; Han et al., 2018). In calcification conditions, AFBs express osteogenic proteins, such as OCN, ALP, Msx2, and CBFα1, similar to VSMCs (Simionescu et al., 2007; Lai et al., 2012).

While these two different cell types have various roles in the vessel, we hypothesized they would utilize RAGE-associated signaling proteins to respond to pathological calcification conditions. This manuscript aims to elucidate the role of RAGE-dependent signaling mechanisms in T2DM-mediated VC in both VSMCs and AFBs under calcification conditions. *In vitro* cell culture methods were used to simulate VC in VSMCs and AFBs independently to determine the impact of a gain or loss of RAGE in non-diabetic and diabetic VSMCs and AFBs. We found the absence of RAGE ameliorated calcification in VSMCs, but not in AFBs. This work will contribute to current knowledge by elucidating the impact of RAGE in an *in vitro* cell culture of two intersecting and prevalent health conditions.

## Materials and Methods

### Animal Model

The subsequent studies utilized genetically diabetic male mice (BKS.Cg-*Dock7*^*m*^+/+*Lepr*^*db*^/J; Jackson Labs; JAX# 00642) (Leiter and Chapman, 1994; Chen et al., 1996; Chua et al., 1996; Lee et al., 1996). The db/db mouse contains a point mutation in the leptin receptor (Lepr) that renders it insensitive to leptin. The heterozygous db/wt (C57BLKS/J *Dock7*^*m*^+/+*Lepr*^*db*^ heterozygote from the colony; Jackson Labs; JAX# 000662; non-diabetic) littermate mice were the lean control group, and they cannot be distinguished morphologically or physiologically from wild-type mice. Non-diabetic (db/wt, non-db, *n* = 124) and diabetic (db/db, db, *n* = 69) mice were utilized for this study. The db/db mouse was crossed with the RAGE knockout mouse (RKO) to generate the following mice for the studies outlined in this manuscript: non-diabetic RAGE knockout (db/wt^*RKO*^, non-db RKO, *n* = 94) and diabetic RAGE knockout (db/db^*RKO*^, db RKO, *n* = 29) (Schwenk et al., 1995; Constien et al., 2001; Lee and Taketo, 2001; Liliensiek et al., 2004; Burr and Stewart, 2020). To generate RKO mice, normal C57Bl/6 mice bred with Cre deleter mice where the Cre/*loxP* recombination system allowed for deleting exons 2–7 of the RAGE gene. This deletion renders the receptor non-functional through a global mRNA knockout of RAGE. The lack of RAGE exons 2–7 confirmed an EGFP reporter gene’s insertion as a PCR positive control for RAGE deletion (Schwenk et al., 1995; Constien et al., 2001; Lee and Taketo, 2001; Liliensiek et al., 2004; Burr and Stewart, 2020). Constien et al. (2001) generated the RAGE knockout line presented in this manuscript and in their publication, and they demonstrated the global knockout of RAGE through immunofluorescence and flow cytometry. Liliensiek et al. (2004) and Perkins et al. (2019) also utilize the global RAGE knockout to study different pathologies of the RKO mouse. Animals were euthanized at 16 weeks of age due to the presence of over diabetes and significant levels of AGEs (Kobayashi et al., 2000). Also, Boström et al. (2011) found significant levels of bone protein expression and visual calcification in the aorta in 20 week old db/db animals. Raaz et al. (2015) also showed that the db/db mouse experienced significantly increase aortic stiffness after 10 weeks of age. Animals were group-housed in an AAALAC-approved animal facility following the National Institutes of Health “Guide for the Care and Use of Laboratory Animals.” Mice experienced a 12 h/12 h light/dark cycle, and food and water were *ad libitum*. The University of Mississippi Animal Care and Use Committee (IACUC protocol number 20-017) approved all animal usage protocols.

### Primary Murine Vascular Smooth Muscle Cell (VSMCs) Isolation and Culture

CO_2_ asphyxiation, followed by cervical dislocation, was performed to euthanize the animals. Bodyweight and non-fasting blood glucose measurements were taken at the time of euthanasia, followed by removing the thoracic aorta (Supplementary Table 1). The same animal provided tissue for both VSMC and AFB isolations as described below. The adventitial layer was separated from the medial layer, and the medial layer was placed in a collagenase-elastase digestion solution [1950 U/A of collagenase type 2 enzyme (Worthington Biochemical), 11.275 units of elastase enzyme activity (Worthington Biochemical), 0.004% Trypsin (Corning), and 10 mL of High-Glucose Dulbecco’s Modified Eagles Medium (HG-DMEM, 4.5 g/L glucose; Corning)] to isolate the cells of the medial and intimal aortic tissue. Tissue pieces and enzymatic digestion solution were maintained in a water-jacketed spinner flask under constant agitation for 45 min. A 100 μm cell strainer was used to filter the tissue-collagenase-elastase solution and 5 mL of HG-DMEM with 30% heat-inactivated Fetal Bovine Serum (FBS) (Innovative Research) was added to neutralize the enzymes. The cell mixture was centrifuged at 225 × *g* for 10 min, and then VSMCs were resuspended in HG-DMEM [14.3 mM NaHCO_3_, 15 mM HEPES, 15% FBS, 2% L-glutamine (Corning), 2X Primocin^TM^ (Invivogen), and Clonetics ^®^ Smooth Muscle Growth Media-2 SingleQuots (Lonza)]. The resuspended cell solution was placed at 37°C at 5% CO_2_ until completion of digestion. The remaining tissue was removed from the strainer and placed in a collagenase digestion solution [100 U/mL type 2 collagenase, 0.1% trypsin (Gibco), and HG-DMEM] in a water-jacketed spinner flask under constant agitation for 30 min. The collagenase digestion was filtered through a 100 μm cell strainer and combined with the previously centrifuged cell solution. All solutions were centrifuged at 225 × *g* for 10 min and then subsequently plated on 100 μg/mL PureCol^®^ collagen-coated (Advanced Biomatrix) 60 mm plates in VSMC HG-DMEM. Twenty-four hours after plating, the cells were washed with appropriate glucose media according to genotype [i.e., HG-DMEM for diabetic and Low Glucose-DMEM (LG-DMEM, 1 g/L glucose- euglycemic media; Corning) for non-diabetic]. VSMCs were maintained in LG-DMEM or HG-DMEM (14.3 mM NaHCO_3_, 15 mM HEPES, 15% FBS, 2% L-glutamine, 2X Primocin^TM^, and Clonetics ^®^ Smooth Muscle Growth Media-2 SingleQuots). All studies used cells at P1 for baseline characterization and P2 for signaling experiments. The purity of the cultures (>90–95%) was confirmed by positive staining for the VSMC-specific marker, α-Smooth Muscle Actin (Sigma-Aldrich, St. Louis, MO, United States, A2547). Aortas from 3 to 4 mice were used per isolation. Data from 6 to 9 separate isolations were collected per genotype. VSMCs cultures were found to be pure with no endothelial cell presence (Supplementary Figure 1).

### Primary Murine Adventitial Fibroblast (AFBs) Isolation and Culture

The same adventitial layer removed for VSMC isolation was utilized for AFBs isolations. The same animal provided tissue for both VSMC and AFB isolations. The minced adventitial tissue was placed in a water-jacketed spinner flask under constant agitation for 10 min. The tissue-collagenase solution was filtered through a 100 μm cell strainer, and 5 mL of HG-DMEM with 30% of FBS was added to neutralize collagenase. The neutralized digestion solution was centrifuged at 225 × *g* for 10 min, and then AFB HG-DMEM (14.3 mM NaHCO_3_, 15 mM HEPES, 15% FBS, 2% L-glutamine, and 2X Primocin^TM^ ) was added to the cell solution. Cells were resuspended in AFB HG-DMEM and placed at 37°C at 5% CO_2_. The remaining tissue was removed from the strainer, placed in fresh collagenase digestion solution, and agitated for 10 min. Subsequent digestions were added to the previous, stored at 37°C at 5% CO_2_, and the digestion was continued until no tissue was left. All solutions were centrifuged at 225 × *g* for 10 min and then subsequently plated in AFB HG-DMEM. Twenty-four hours after plating, the cells were washed with appropriate glucose media according to genotype (i.e., HG-DMEM for diabetic and LG-DMEM for non-diabetic). AFBs were maintained in AFB LG-DMEM or HG-DMEM. All studies used cells at P1 for baseline characterization and P2 for signaling experiments. The purity of the cultures (>90–95%) was confirmed by positive staining for the AFB-specific marker, vimentin (Santa Cruz Biotechnology, sc-32322) (Supplementary Figure 1). Aortic adventitia from 3 to 4 mice was used per isolation, and data from 6 to 9 separate isolations were collected per genotype.

### Pharmacological Treatment of Cells

Cells were passaged until P2, and when confluent, cells were washed in 1X sterile PBS. Experiments were conducted in 2% FBS genotype appropriate DMEM to reduce cell division. Pertinent groups were treated with 0.5 mg/mL albumin (glycated, human, AGEs, Sigma) and/or 3 mM inorganic phosphate at the start of each study and reapplied on day 3. Studies concluded on day 7 (Tanikawa et al., 2009; McArthur et al., 2017). For calcification experiments, cells were seeded onto 96-well plates (Eppendorf) with matched wells for calcification and cell number, and for protein experiments, cells were seeded onto 60-mm dishes (Corning).

### Calcification Quantitation

Cells were washed in 1X PBS twice, and then 250 μL 0.6 N HCl was placed on the cells for 24 h. The HCl supernatant was collected after 24 h at room temperature, and the calcium content was measured with the Calcium Colorimetric Kit (MAK-022; Sigma-Aldrich). Cells were fixed with 4% PFA on the matched wells for 10 min and then washed with 1X PBS 2 times for 5 min each wash. 1:1000 DAPI in 0.01% Triton X-100 in 1X PBS was placed on the cells overnight at 4°C. After incubation, cells were washed in 1X PBS twice for 5 min each wash and imaged with high content analysis Nikon Ti2-E microscope using a high-speed PCOS camera. Images obtained were analyzed with high content analysis software with automated cell counting, cell growth analysis, proximity analysis, and Nikon propriety JOBS analysis (NIS-Elements, RRID:SCR_014329). Calcium content (μg) was normalized to cell number. Each experimental replicate is representative of 3 experimental repeats for each group.

### Alizarin Red Staining

Cells were washed in 1X PBS and fixed for 10 min at RT in 4% PFA. PFA was removed, and cells were washed briefly in diH_2_O 3 times for 5 min each wash. Cells were stained in 2% Alizarin Red in diH_2_O for 10 min. Cells were washed three times for 5 min in diH_2_O and then imaged at 4X on Zeiss Primovert with Axiocam ERc 5 s.

### Protein Analysis

Cells were lysed using 100 μL of modified Hunter’s Buffer [1% Triton X-100, 75 mM NaCl, 5 mM Tris (pH 7.4), 0.5 mM orthovanadate, 0.5 mM EDTA, 0.5 mM EGTA, 0.25% NP-40 and Halt-Protease Inhibitor Cocktail (100X; Thermo Scientific)]. On ice, cells were scraped, and lysates were collected. Cell lysates were sonicated for 5-s bursts until disrupted. Lysates were centrifuged for 15 min at 32,000 × *g* at 4°C. The supernatant was transferred to new 1.5 mL centrifuge tubes and stored at -80°C until further analysis. Protein concentration for each sample was determined using the bicinchoninic acid (BCA) assay (Pierce Biotechnology) according to the manufacturer’s instructions. The proteins were separated based on their molecular weight on a 12% SDS-PAGE gel. The proteins were transferred to methanol-activated Immobilon-P PVDF membrane (Millipore Sigma). The membrane was blocked from non-specific binding in either 5% milk or 5% BSA diluted in TBS-T (50 mM Tris-Base, 100 mM NaCl, and pH 7.4 with 0.001% Tween-20). Primary antibodies were placed on the membrane in either 5% milk or 5% BSA diluted in TBS-T. The following primary antibodies were utilized: RAGE (1:400; Santa Cruz Biotechnology, sc-365154, RRID:AB_10707685), α-SMA (1:1,000; Sigma-Aldrich, A2547, RRID:AB_476701), vimentin (1:1,000; Cell Signaling, 5741, RRID:AB_10695459), Osteopontin (OPN; 1:400; Abcam, ab8448, RRID:AB_306566), Toll-like Receptor 4 (TLR4; 1:400; Santa Cruz Biotechnology, sc-293072, RRID:AB_10611320), phosphorylated ERK 1/2 (1:400; Santa Cruz Biotechnology, sc-7383, RRID:AB_627545), total ERK 1 (1:400; Santa Cruz Biotechnology, sc-271269, RRID:AB_10611091), total ERK 2 (1:400; Santa Cruz Biotechnology, sc-1647, RRID:AB_627547), phosphorylated NF-κB p65 (1:400; Santa Cruz Biotechnology, sc-136548, RRID:AB_10610391), phosphorylated p38 MAPK (1:1,000; Cell Signaling, 9211, RRID:AB_331641), total p38 MAPK (1:1,000; Cell Signaling, 9212, RRID:AB_330713), Superoxide Dismutase 2 (1:400; Santa Cruz Biotechnology, sc-133134, RRID:AB_2191814), and β-tubulin (1:400; Santa Cruz Biotechnology, sc-398937) as a loading control (Bassi et al., 2017; Li et al., 2020). Primary antibodies were incubated with the membrane overnight at 4°C. The membrane was washed with TBS-T 5 times for 5 min each wash and then incubated at room temperature with a secondary antibody conjugated to Horseradish peroxidase (HRP) (Santa Cruz Biotechnology). The membrane was washed with TBS-T 5 times for 5 min each wash and then incubated in Pierce enhanced chemiluminescent substrates (Thermo Fisher Scientific) for 2 min. The membrane was visualized with the iBRIGHT imaging system, and densitometric analysis on bands was performed with NIH Image J software.

### Hydrogen Peroxide (H_2_O_2_) Assay

Protein samples were isolated as described above. OxiSelect^TM^ Hydrogen Peroxide Colorimetric Assay Kit (Cell Biolabs, Inc., STA-844) was utilized. Twenty five microliter of each sample and 25 μL of kit provided diluent were added to each sample well. Manufacturer instructions were followed for the duration of the kit and analysis of results.

### Statistical Analysis

Data presented are mean ± standard error of the mean (SEM) where each experiment was performed a minimum of *n* ≥ 3. A one-way or two-way ANOVA was performed with Graph Prism software, version 8.4.3. Each type of ANOVA was selected based on applicable data. All figures were analyzed with a Fisher’s LSD *post hoc*. This analysis method was performed because the analysis’s overall *p*-value must be significant for the *post hoc* to detect significance. Fisher’s LSD also does not consider comparisons between other groups, and each comparison stands alone. *Post hoc* results on graphs are focused on groups of interest, and only values of *p* < 0.05 were considered statistically significant.

## Results

### Addition of Exogenous AGEs Altered RAGE-Mediated VSMC Calcification

**FIGURE 1:**
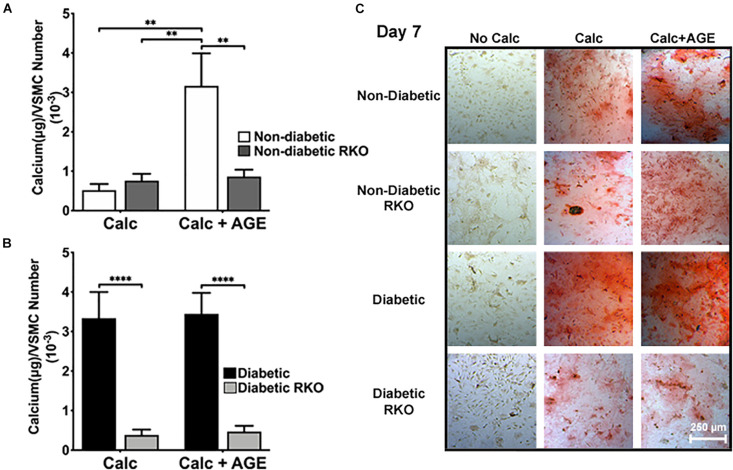
Vascular Smooth Muscle Cell (VSMC) calcification is affected by the presence of AGE-mediated RAGE signaling. Primary mouse VSMCs were isolated from non-diabetic and diabetic aortas with or without the presence of RAGE. VSMCs were plated onto 96 well plates and treated with 3 mM P_*i*_ with or without AGEs (0.5 mg/mL) for 7 days. **(A,B)** Calcium (μg) was normalized to cell number and graphed as mean ± SEM with *n* = 6–9 of independent replicates. **(C)** Alizarin red staining indicated calcification by the red staining. (4X and scale bar = 200 μM) Statistical analysis consisted of a one-way ANOVA and a Fisher’s LSD test *post hoc* (**p < 0.05, ****p < 0.0001).

AGE-mediated RAGE signaling induced vascular smooth muscle cell calcification. Non-diabetic cells showed a significant increase in calcification with the addition of AGEs (Figures 1A,C). There were no considerable increases in calcification with the addition of AGEs in diabetic cells, while non-diabetic cells with AGEs increased calcification to diabetic levels (Figures 1B,C). In contrast, the loss of RAGE in both non-diabetic and diabetic RKO VSMCs exhibited no changes in calcification, and with the addition of AGEs, calcification levels did not change (Figures 1A–C). VSMCs appeared to undergo RAGE-dependent calcification.

### VSMCs Differentiated From a Contractile Phenotype to an Osteogenic Phenotype

**FIGURE 2:**
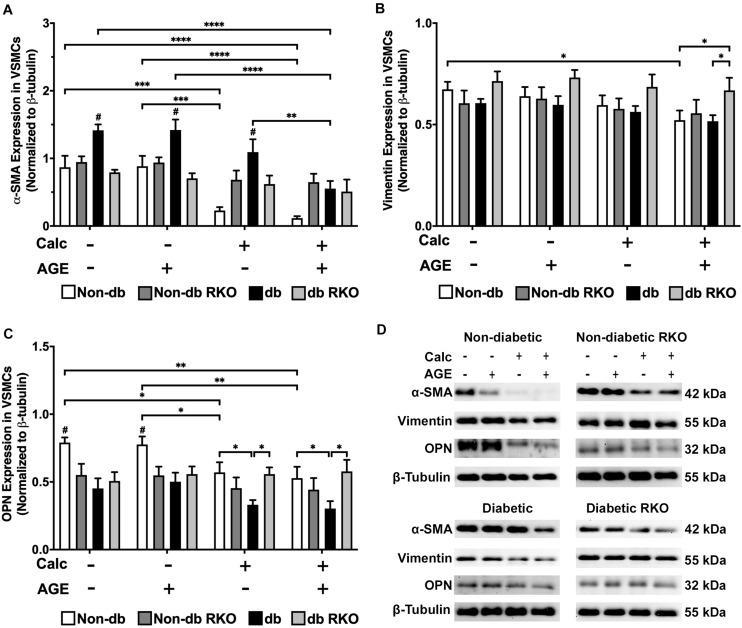
Vascular smooth muscle cells respond to calcification by differentiating away from the contractile phenotype and toward a different cell type. Protein expression of α-SMA (42 kDa; **A**), vimentin (55 kDa; **B**), and OPN (32 kDa; **C**) was quantified. Non-diabetic, non-diabetic RKO, diabetic, and diabetic RKO primary VSMCs were isolated and treated with 3 mM P_*i*_ with or without AGEs (0.5 mg/mL) for 7 days. Protein levels were normalized to β-tubulin **(D)** and graphed as mean ± SEM with *n* = 6–9 of independent replicates. Statistical analysis consisted of a two-way ANOVA and a Fisher’s LSD test *post hoc* (^#^*p* < 0.05 toward all other groups in treatment group, *p < 0.05, **p < 0.01, ***p < 0.001, ****p < 0.0001).

Protein expression analysis revealed that diabetic VSMCs have a higher RAGE expression than non-diabetic VSMCs (Supplementary Figure 2A,B). With calcification treatment and the addition of exogenous AGEs, RAGE expression levels significantly decreased in non-diabetic cells. No changes were observed in RAGE expression in diabetic cells with AGE treatment (Supplementary Figure 2A,B). VSMC phenotype marker, α-smooth muscle actin (SMA), was significantly decreased in calcified non-diabetic cells. Calcification and exogenous AGE treatments further reduced α-SMA expression in comparison to non-calcification groups (Figure 2A,D). Diabetic cells showed significantly reduced expression of α-SMA with the addition of exogenous AGEs and calcification treatment. Untreated diabetic cells had significantly higher expression of α-SMA than other untreated genotypes (Figure 2A,D). Non-diabetic RKO and diabetic RKO did not show significant changes in α-SMA due to calcification treatments (Figure 2A,D). Non-diabetic cells had a loss of vimentin with calcification and AGEs (Figure 2B,D). OPN expression significantly decreased in non-diabetic VSMCs with calcification and AGEs treatment (Figure 2C,D). Non-diabetic RKO and diabetic RKO VSMCs showed no OPN expression changes (Figure 2C,D). Overall, protein expression indicated a loss of VSMC contractile to osteogenic phenotype in a RAGE-dependent manner.

### Calcification and Exogenous AGE Treatments of VSMCs Increased Protein Expression of Signaling Molecules Associated With the AGE/RAGE Cascade

**FIGURE 3:**
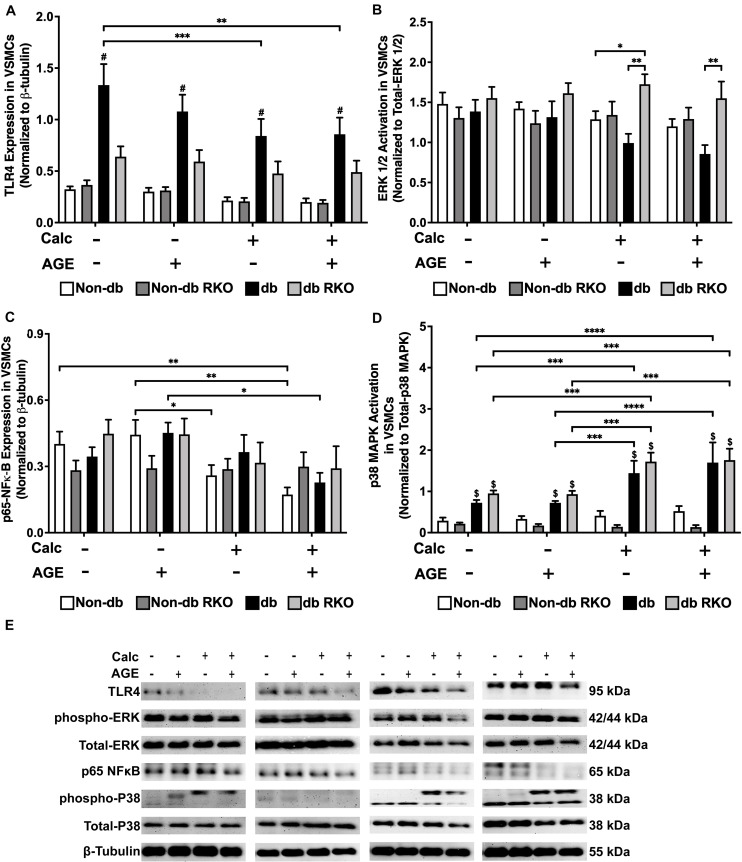
Diabetic vascular smooth muscle cells utilize RAGE signaling through the phospho-p38 MAPK-RAGE axis. Protein expression of TLR4 (95 kDa; **A**), activated ERK 1/2 (42/44 kDa; **B**), phosphorylated-p65-NF-κB (65 kDa; **C**), and activated p38-MAPK (38 kDa; **D**) was quantified. Non-diabetic, non-diabetic RKO, diabetic, and diabetic RKO primary VSMCs were isolated and treated with 3 mM P_*i*_ with or without AGEs (0.5 mg/mL) for 7 days. Protein levels were normalized to β-tubulin **(E)** and graphed as mean ± SEM with *n* = 6–9 of independent replicates. Statistical analysis consisted of a two-way ANOVA and a Fisher’s LSD test *post hoc* (^$^*p* < 0.05 toward non-diabetic cell types in treatment group, *p < 0.05, **p < 0.01, ***p < 0.001, ****p < 0.0001).

Diabetic VSMCs showed an elevated expression of TLR4 compared to other genotypes, but protein expression significantly declined with calcification and AGEs treatment (Figure 3A,E). There were no relevant changes in ERK 1/2 activation with treatment in VSMCs (Figure 3B,E). Calcification treatment in non-diabetic VSMCs significantly decreased phosphorylated-NF-κB expression, and the addition of AGEs with calcification caused a sharper decrease in phosphorylated-NF-κB expression in these cells when compared to no calcification and AGEs treatment (Figure 3C,E). Diabetic VSMCs showed the same expression pattern as non-diabetic cells, but only calcification and AGEs led to significant changes in these cells when compared to no calcification and AGEs treatment (Figure 3C,E). RKO cells did not show differences in phosphorylated-NFκB expression (Figure 3C,E). Phosphorylated p38 MAPK activation was not changed in non-diabetic and non-diabetic RKO VSMCs, although there was an upward trend in p38 MAPK activation with calcification and AGEs in non-diabetic cells (Figure 3D,E). In contrast, diabetic and diabetic RKO VSMCs showed significant increases in p38 MAPK activation with the addition of calcification and AGEs (Figure 3D,E). Protein evidence indicated AGE/RAGE-associated signaling proteins, such as ERK 1/2, p-NF-κB, and p-p38 MAPK, were altered to possibly promote a pattern of oxidative stress in response to calcification and exogenous AGE treatment.

### Calcified Non-diabetic VSMCs Had Increased H_2_O_2_ Production Without Concomitant Changes in SOD-2 Protein Expression

**FIGURE 4:**
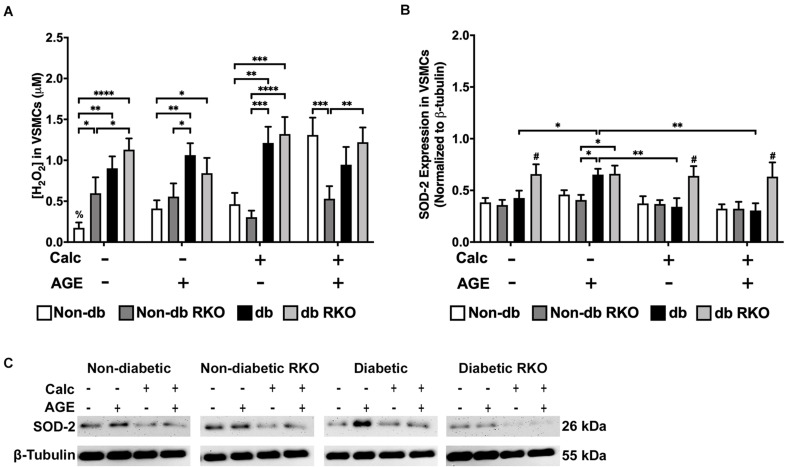
The concentration of H_2_O_2_ was increased due to calcification in VSMCs and SOD-2 expression was increased due to AGEs in diabetic VSMCs. The concentration of H_2_O_2_ was measured colorimetrically **(A)**. Protein expression of SOD-2 (26 kDa; **B**) was quantified. Non-diabetic, non-diabetic RKO, and diabetic, and diabetic RKO primary VSMCs were isolated and treated with 3 mM P_*i*_ with or without AGEs (0.5 mg/mL) for 7 days. Protein levels were normalized to β-tubulin **(C)** and graphed as mean ± SEM with *n* = 6–9 of independent replicates. Statistical analysis consisted of a two-way ANOVA and a Fisher’s LSD test *post hoc* (^#^*p* < 0.05 toward all other groups in treatment group, ^%^*p* < 0.05 toward all other treatments within genotype, *p < 0.05, **p < 0.01, ***p < 0.001, ****p < 0.0001).

Non-diabetic VSMCs had significantly increased H_2_O_2_ production with exogenous AGEs and calcification, separately, and when treatments were combined, H_2_O_2_ production significantly increased over no calcification groups (Figure 4A). Additionally, diabetic VSMCs had significantly more H_2_O_2_ production than non-diabetic cell types across all treatments, except when non-diabetic cells were treated with combined calcification and exogenous AGEs. However, these cells reached diabetic levels. H_2_O_2_ production in RKO cells remained unchanged (Figure 4A). SOD-2 expression did not change with treatments in either non-diabetic and non-diabetic RKO cell genotypes. Diabetic VSMCs had significantly more SOD-2 expression with the addition of AGEs alone. Diabetic RKO cells have a significantly higher expression level of SOD-2 across all treatment groups compared to both non-diabetic and non-diabetic RKO cells (Figure 4B,C). Higher H_2_O_2_ production coincided with calcification and AGE treatments; however, these changes were not matched by changes in SOD-2 expression.

### Loss of RAGE Expression Did Not Change AFBs Calcification

**FIGURE 5:**
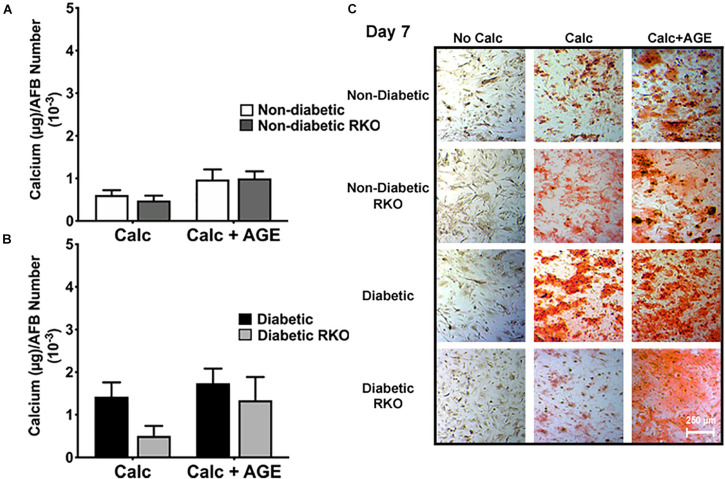
Adventitial fibroblasts (AFBs) calcification is not affected by the presence of RAGE. Primary mouse AFBs were isolated from non-diabetic and diabetic aortas with or without the presence of RAGE. AFBs were plated onto 96 well plates and treated with 3 mM P_*i*_ with or without AGEs (0.5 mg/mL) for 7 days. **(A,B)** Calcium (μg) was normalized to cell number and graphed as mean ± SEM with *n* = 6–9 of independent replicates. **(C)** Alizarin red staining indicated calcification by the red staining. (4X and scale bar = 200 μM) Statistical analysis consisted of a one-way ANOVA and a Fisher’s LSD test *post hoc*.

The lack of RAGE presence did not significantly change AFBs calcification in all treatment groups and genotypes (Figure 5A-C). Non-diabetic RKO AFBs calcified in similar patterns compared to non-diabetic AFBs with calcification and AGE treatments. These patterns suggested that non-diabetic AFBs calcification was not dependent on AGE-mediated RAGE signaling (Figure 5A). Also, with the addition of AGEs to diabetic cells, no further calcification changes were observed (Figure 5B). There was a slight increase in the diabetic RKO AFBs in calcification with AGEs, which was the same trend observed in the non-diabetic cells (Figure 5B); however, these changes were not significant. These findings suggested there may be another signaling pathway responsible for calcification in AFBs.

### AFB Phenotype Markers Altered in Response to Calcification and Exogenous AGE Treatments

**FIGURE 6:**
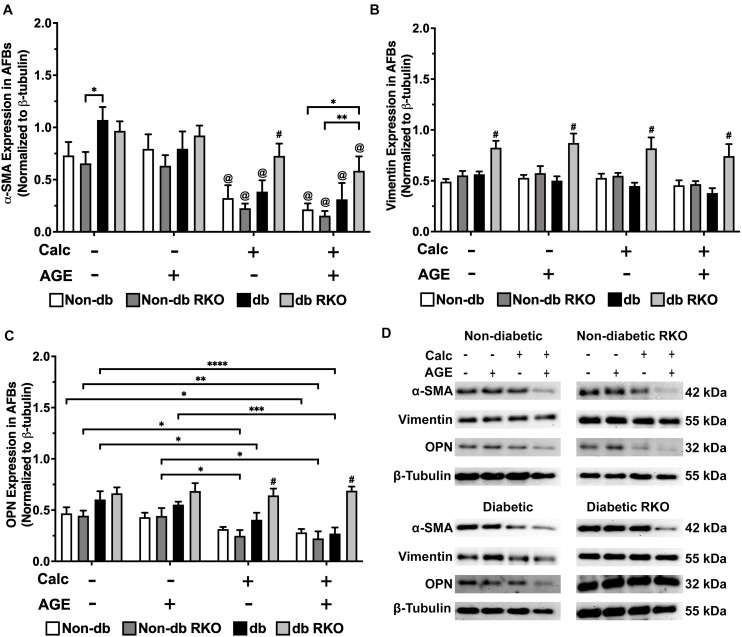
Adventitial fibroblasts respond to calcification by a loss of phenotype markers. Protein expression of α-SMA (42 kDa; **A**), vimentin (55 kDa; **B**), and OPN (32 kDa; **C**) was quantified. Non-diabetic, non-diabetic RKO, diabetic, and diabetic RKO primary AFBs were isolated and treated with 3 mM P_*i*_ with or without AGEs (0.5 mg/mL) for 7 days. Protein levels were normalized to β-tubulin **(D)** and graphed as mean ± SEM with *n* = 6–9 of independent replicates. Statistical analysis consisted of a two-way ANOVA and a Fisher’s LSD test *post hoc* (^#^*p* < 0.05 toward all other groups in treatment group, ^@^*p* < 0.05 toward no calcification treatments within genotype, *p < 0.05, **p < 0.01, ***p < 0.001, ****p < 0.0001).

In non-diabetic AFBs, there were no significant changes in RAGE expression; however, in diabetic AFBs, there were losses in RAGE expression with calcification and AGE treatments (Supplementary Figure 2C,D). α-SMA expression, a myofibroblast marker, was significantly reduced due to calcification and AGE treatments in all genotypes (Figure 6A,D). Vimentin, a fibroblast marker, expression remained relatively low and unchanged in all genotypes except for diabetic RKO, where it was significantly higher than all other genotypes within a treatment group (Figure 6B,D). OPN expression was decreased in all genotypes due to calcification treatment except for diabetic RKO cells. Diabetic AFBs showed a slight reduction in OPN with AGE treatment, whereas non-diabetic and non-diabetic RKO cells did not respond to AGE addition (Figure 6C,D). AFBs lost expression of α-SMA and OPN due to calcification treatment, while vimentin remained the same in all genotypes except for diabetic RKO.

### Calcification and Exogenous AGE Treatments Caused Differential Signaling Responses in AFBs

**FIGURE 7:**
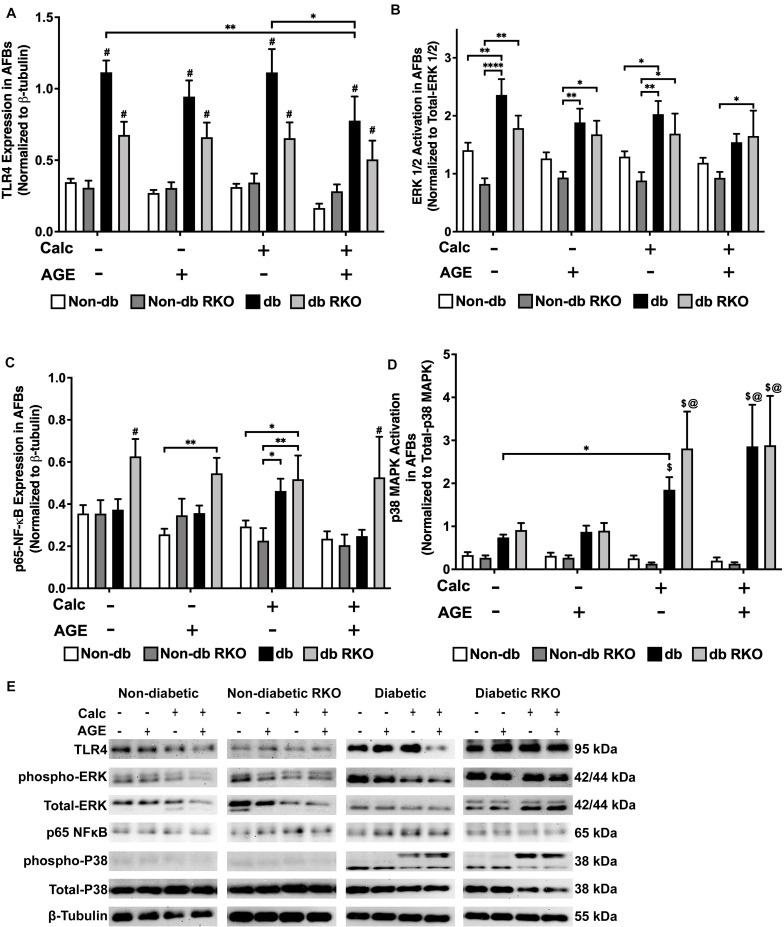
Adventitial fibroblasts may signal through the p38 MAPK/RAGE-axis in response to calcification. Protein expression of TLR4 (95 kDa; **A**), activated ERK 1/2 (42/44 kDa; **B**), phosphorylated-p65-NF-κB (65 kDa; **C**), and activated p38-MAPK (38 kDa; **D**) was quantified. Non-diabetic, non-diabetic RKO, diabetic, and diabetic RKO primary AFBs were isolated and treated with 3 mM P_*i*_ with or without AGEs (0.5 mg/mL) for 7 days. Protein levels were normalized to β-tubulin **(E)** and graphed as mean ± SEM with *n* = 6–9 of independent replicates. Statistical analysis consisted of a two-way ANOVA and a Fisher’s LSD test *post hoc* (^#^*p* < 0.05 toward all other groups in treatment group, ^$^*p* < 0.05 toward non-diabetic cells in treatment group, ^@^*p* < 0.05 toward no calcification treatments within genotype, *p < 0.05, **p < 0.01).

Western blot analysis detected no changes in TLR4 expression in other genotypes. However, TLR4 expression was higher in diabetic cells than all other cell types, and with the combined calcification and exogenous AGE treatment, there was a significant loss in TLR4 expression in diabetic AFBs (Figure 7A,E). There were no significant changes in phospho-ERK 1/2 activation due to either calcification, exogenous AGE, or combined treatments in any genotype, but treatment with AGEs alone caused a slight but insignificant decrease phospho-ERK 1/2 activation in diabetic AFBs. However, diabetic cells did have significantly higher phospho-ERK 1/2 expression levels than non-diabetic cell AFBs (Figure 7B,E). Phosphorylated-p65-NF-κB expression showed no changes in non-diabetic or non-diabetic RKO cells. Diabetic AFBs phosphorylated-p65-NF-κB expression slightly increased with calcification. Phosphorylated-NFκB expression remained the same with calcification and AGE treatment in all genotypes except for diabetic RKO. Diabetic RKO cells showed no changes, but their overall phosphorylated-NF-κB expression rose above other genotypes in the same treatment group (Figure 7C,E). Neither non-diabetic nor non-diabetic RKO AFBs displayed any changes in p38 MAPK activation. Diabetic and diabetic RKO cells had significant increases in p38 MAPK activation with calcification and exogenous AGE treatment (Figure 7D,E). Both diabetic cell types showed p38 MAPK activation was significantly higher in both calcified treatment groups (Figure 7D,E). AFBs had a significant increase in p38-MAPK activation compared to the other signaling molecules investigated.

### Calcification and Exogenous AGE Treatment of AFBs Led to Increased H_2_O_2_ Production and SOD-2 Expression

**FIGURE 8:**
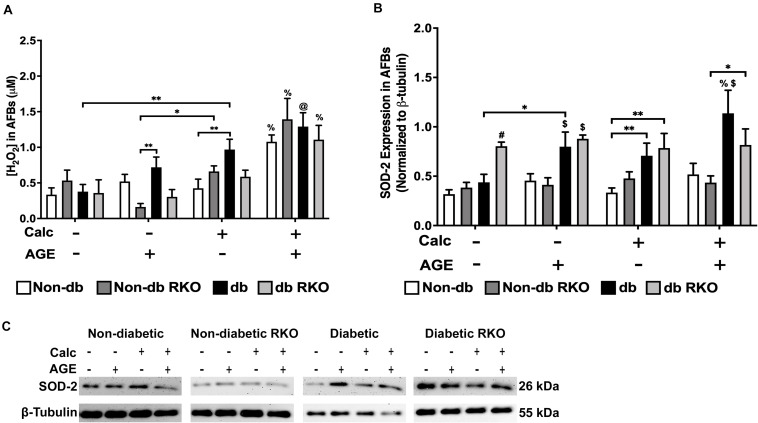
The concentration of H_2_O_2_ and SOD-2 expression was increased due to calcification and AGEs in diabetic AFBs. The concentration of H_2_O_2_ was measured colorimetrically **(A)**. Protein expression of SOD-2 (26 kDa; **B**) was quantified. Non-diabetic, non-diabetic RKO, and diabetic, and diabetic RKO primary AFBs were isolated and treated with 3 mM P_*i*_ with or without AGEs (0.5 mg/mL) for 7 days. Protein levels were normalized to β-tubulin **(C)** and graphed as mean ± SEM with *n* = 6–9 of independent replicates. Statistical analysis consisted of a two-way ANOVA and a Fisher’s LSD test *post hoc* (^#^*p* < 0.05 toward all other groups in treatment group, ^%^*p* < 0.05 toward all other treatments within genotype, ^$^*p* < 0.05 toward non-diabetic cell types in treatment group, ^@^*p* < 0.05 toward no calcification treatments within genotype, *p < 0.05, **p < 0.01).

Combined calcification and exogenous AGE treatment resulted in all genotypes having significantly higher H_2_O_2_ production than untreated and calcification and exogenous AGE treatments alone in all AFBs genotypes (Figure 8A). SOD-2 expression in treated groups remained relatively unchanged and similar to untreated groups in non-diabetic, non-diabetic RKO, and diabetic RKO cells. However, diabetic RKO cells did have significantly higher SOD-2 expression levels in untreated AFBs. Significantly more SOD-2 expression observed in diabetic AFBs was in response to calcification and exogenous AGE treatment (Figure 8B,C). AFBs produce more H_2_O_2_ due to treatment with calcification and AGEs.

## Discussion

In *in vivo* physiological conditions, VSMCs and AFBs experience a very similar microenvironment. These two cell types reside in different vessel layers, but imbalances in minerals and hyperglycemia are still present throughout the vessel architecture. The VSMCs of the medial layer are one of the most studied cell types from the aorta, followed by the intimal layer endothelial cells, while the AFBs remain mostly uncharacterized. Therefore, in this study, we sought to compare the individual VSMCs and AFBs response to calcification and to exogenous AGE treatments in an *in vitro* VC model. Diabetic cells were maintained in high glucose to mimic a hyperglycemic environment as found in diabetic patients, and non-diabetic cells were grown in low glucose, similar to euglycemic levels. This study also used cells from diabetic and non-diabetic RAGE knockout mice and exogenous AGE treatments to elucidate the impact of AGE/RAGE signaling on phenotype changes, and RAGE-dependent signaling mechanisms previously demonstrated to facilitate VC. Overall, we found that the presence of RAGE mediated VSMC diabetes-mediated calcification while AFBs responses were not RAGE dependent.

Under calcification conditions, non-diabetic VSMCs presented with higher amounts of calcification after the addition of exogenous AGES. This finding indicates VSMCs were dependent upon AGE/RAGE signaling to increase calcification levels. Tanikawa et al. (2009) demonstrated that calcification of human aortic smooth muscle cells increased with the addition of AGEs. Also, Ren et al. (2009) as well as Wei et al. (2013) had similar findings using rat aortic smooth muscle cells. In our study, we found non-diabetic VSMC calcification levels were equal to those observed in diabetic VSMCs when calcification and exogenous AGE treatments were applied. While there were no increases were documented in calcification levels with the addition of exogenous AGEs in diabetic VSMCs, this finding may be due to increased basal levels of RAGE expression and circulating AGEs found in diabetics. Bao et al. (2020) found that diabetic patients had more vascular RAGE expression than their control, non-diabetic counterparts. RKO VSMC type calcification was not affected by the addition of AGEs, which points to AGE/RAGE signaling being involved in VSMC calcification. Additionally, RKO VSMCs had very little calcification present, as VSMC calcification pointed to being governed by RAGE-dependent mechanisms. We found AFBs calcification did not appear dependent upon RAGE. All genotypes responded to AGE treatment by slightly increased calcification levels. Therefore, we believe RAGE may not be the only AGE-mediated receptor present on AFBs capable of influencing calcification. According to Bierhaus et al., AGEs are capable of stimulating the vessel in various ways, including activation of NF-κB and oxidative stress pathways, deposition of the collagenous matrix, and increasing DNA mutation rate (Bierhaus et al., 1998). To understand the calcification mechanisms, we sought to analyze RAGE-dependent phenotype markers and signaling proteins in both cell types under calcification conditions with and without exogenous AGEs.

VSMCs have been demonstrated to lose their primary phenotype marker, α-SMA, under calcification conditions (Steitz et al., 2001; Speer et al., 2010; Shanahan et al., 2011; Durham et al., 2018). Our findings showed similar significant decreases in α-SMA in non-diabetic VSMCs after calcification and AGE treatments, and these observations occurred diabetic VSMCs but to a lesser extent. However, in non-diabetic and diabetic RKO VSMCs, α-SMA protein expression remained near untreated levels despite calcification and AGE treatments, which demonstrated the presence of RAGE was required to cause phenotype changes in VSMC. This also points to that when RAGE is lost, VSMCs do not respond to calcification stimuli. When added to the data showing a loss of vimentin was observed in non-diabetic VSMCs under calcification conditions and exogenous AGE treatments, the cells could be undergoing a loss of the VSMC phenotype and undergoing differentiation to a different cell type in an AGE/RAGE-dependent manner. These VSMC cell types may include osteochondrogenic-like cells, foam cells, or a synthetic phenotype, hence demonstrating the plasticity of VSMCs (Bobryshev, 2005; Sinha et al., 2014; Durham et al., 2018; Bao et al., 2020). OPN expression changes have also been linked to VSMC calcification and phenotype changes (Jono et al., 2000; Giachelli et al., 2005; Speer et al., 2005; Noda and Denhardt, 2008; Scatena et al., 2007; Lok and Lyle, 2019). In our study, we detected decreased cleaved OPN expression in non-diabetic VSMCs, while RKO cells had no change in OPN expression levels, indicating RAGE was also regulating OPN expression and/or its cleavage by matrix metalloproteinases. Literature from other disease models found a correlation between RAGE and OPN (El-Asrar et al., 2011; Palanissami and Paul, 2018). El-Asrar et al. (2011) using diabetic human epiretinal fibrovascular membranes to investigate proliferative diabetic retinopathy (PDR) and proliferative vitreoretinopathy, found a significantly higher expression of RAGE and full-length OPN in blood vessels and cells with active PDR over that from inactive patients. They suggested a correlation between the OPN and RAGE axis existed to promote PDR (El-Asrar et al., 2011). In addition to PDR, cancer impacts RAGE and OPN expression patterns. Palanissami and Paul (2018) reviewed several papers that correlated RAGE and its ligands to the increased expression of OPN in other disease states. Our results differed from the published findings in that we observed changes in cleaved OPN and not the full-length. Agnihotri et al. (2001) demonstrated that the cleaved fragment of OPN that we detect with our antibody was cleaved by MMP-7 and the cleaved fragments have greater adhesion to rat smooth muscle cells and NIH3T3 fibroblasts. Giachelli et al. (2005) conclude that OPN is a potent inhibitor of calcification, but the loss of OPN leads to calcification Therefor with these two findings, our results would suggest that the loss of cleaved OPN that is more adherent to cell types similar to ours would lead to more calcification. Diabetic VSMCs from our study did not show significant responses in OPN expression as a result of increased AGE/RAGE signaling. We believe the increased presence of RAGE may allow for homeostatic balance to be achieved with OPN expression and OPN cleavage. Therefore, RAGE-regulated OPN expression may exist as an equilibrium in the cell, maintaining cleaved OPN at appropriate levels in response to treatments. In contrast, there were no changes in OPN expression in RKO cells. Therefore, the presence of RAGE was required to produce changes in calcification, and these phenotypic changes were only present in both non-diabetic and diabetic VSMCs wildtype for RAGE. Consequently, leading us to hypothesize VSMC calcification was mediated by the AGE/RAGE signaling cascade.

In AFBs, there were significant losses in α-SMA due to calcification. In other stressful situations, fibroblasts express more α-SMA and transition to a myofibroblast or “activated fibroblast” phenotype, characterized by a loss of markers such as vimentin (Li et al., 2015; Han et al., 2018). Our findings would suggest that the AFBs are undergoing a similar phenotype transition. Simionescu et al. suggested fibroblasts can express osteogenic markers such as osteocalcin, alkaline phosphatase, and osteoprotegerin under calcification conditions like that of VSMCs (Simionescu et al., 2007; Pillai et al., 2017). Zorzi et al. (2010) also showed that some AFBs could transition to a macrophage-like phenotype with phagocytic properties and MHC II expression. While there were no changes due to treatments in vimentin expression, there were higher vimentin expression levels maintained in diabetic RKO cells. Kueper et al. (2007) showed AGEs could directly attach to vimentin, preventing vimentin turnover resulting in elevated diabetic RKO expression. From these findings we believe the loss of α-SMA and differences in vimentin compared to RKO AFBs indicate the initiation of a change in AFB phenotype. More work will need to be performed in order to further elucidate these results. In addition to these findings, there were decreased cleaved OPN expression levels in all cell types except diabetic RKO. As outlined above, decreased levels of cleaved OPN would lead to more calcification (Agnihotri et al., 2001; Giachelli et al., 2005). These results suggest that perhaps another signaling molecule besides RAGE may regulate calcification in the non-diabetic, diabetic and non-diabetic RKO AFBs. While AFBs phenotype marker expression indicated AGE-mediated RAGE signaling might not be the sole regulator of AFB calcification, more work is needed to delineate changes as a result of these signaling cascades.

To further explore RAGE signaling and the possibility of an alternate AGE receptor, we accessed protein levels for other signaling molecules known to be associated with the AGE-mediated signaling. Diabetic VSMCs showed increased levels of TLR4 protein expression. We believe these changes may coincide with increased RAGE expression observed in untreated diabetic VSMCs. However, calcification and AGE treatments resulted in a decrease in TLR4 expression in the diabetic VSMCs. Sakaguchi et al. (2011) and Olejarz et al. (2018) suggested that RAGE and TLR4 share similar ligands and have adaptor proteins in common. These cascades can activate some of the same transcription factors, serving as possible targets for pharmacological intervention for diseases, such as atherosclerosis. Atherosclerosis and VC share several signaling components in common (Yahagi et al., 2017). We believe that RAGE and TLR4 are involved in balancing the AGE-induced cellular responses as a result of calcification and exogenous AGE treatments (Ibrahim et al., 2013). Ga̧siorowski et al. (2018) also reported TLR4-RAGE crosstalk was responsible for chronic inflammation in Alzheimer’s disease. Additionally, TLR4 and RAGE can activate and regulate the ERK 1/2 signaling pathway (Soman et al., 2013; Seo et al., 2018). In our study, we showed elevated levels of activated ERK 1/2 signaling in diabetic RKO VSMCs. Zhu et al. (2012) demonstrated elevated levels of ERK 1/2 in Schwann cells after exposure to a hyperglycemic environment. Also, Galvão et al. (2020) showed increased expression of ERK 1/2 in macrophages from diabetic animals to cause dysregulation of a number of pro-inflammatory cytokines. In addition to ERK1/2, we investigated phospho-p65-NF-KB, which was decreased in non-diabetic and diabetic VSMCs under calcification conditions. Lower phospho-p65-NF-KB expression levels could indicate that the molecule was cycling through peaks and troughs of expression via RAGE signaling (Yeh et al., 2001; Yuan et al., 2011; Hu et al., 2012). However, p38 MAPK activation increased due to calcification and exogenous AGE treatments in diabetic VSMCs. These findings correlated with reported studies in the literature demonstrating increased p38 MAPK activation resulted in chronic changes in cytokine profiles altered as a result of calcification and RAGE stimulation (Geraldes et al., 2009; Tanikawa et al., 2009; Giacco and Brownlee, 2010). One finding we noted was the significant increase in p38 MAPK in diabetic RKO cells. The significant increase might be due to the hyperglycemic environment or other signaling factors modulating p38 MAPK, and not RAGE dependent factors, such as JNK, TGF-β, or Rho/ROCK (Zarubin and Han, 2005; Coulthard et al., 2009; Bassi et al., 2017). The changes in the reported signaling factors may depend upon the level of RAGE signaling or the level inclusion of TLR4 signaling mechanisms, which may indicate VSMCs calcification dependence on AGE-mediated signaling.

Like diabetic VSMCs, diabetic AFBs expressed TLR4 at significantly higher protein expression levels than observed in non-diabetic cells. As mentioned previously, RAGE and TLR4 could be involved in balancing many different cellular processes in health and disease (Sakaguchi et al., 2011; Ibrahim et al., 2013; Ga̧siorowski et al., 2018; Olejarz et al., 2018). When downstream signaling proteins were evaluated, ERK 1/2 and p38 MAPK activation levels were higher in diabetic and diabetic RKO AFBs, which previous findings support these observations (Zhu et al., 2012; Galvão et al., 2020). In diabetic RKO AFBs, the observations may be linked to the hyperglycemic-mediated changes or other factors regulating ERK 1/2 and p38 MAPK activation, such as epidermal growth factor receptor (EGFR), oxidative stressors, secreted cytokines, Rac1, or Cdc42 (Zarubin and Han, 2005; Coulthard et al., 2009; Wee and Wang, 2017). P38 MAPK activation was also increased due to calcification in both diabetic cell types, suggesting calcification might be AGE/RAGE independent. Also, both cell types could be utilizing different p38 MAPK modulated pathways without RAGE activation (Zarubin and Han, 2005; Coulthard et al., 2009). Phospho-p65-NF-κB expression significantly increased in diabetic RKO AFBs in all treatment groups, which could be attributed to the hyperglycemic environment or the lack of RAGE allowing for increased expression of phospho-p65-NF-κB through other activation pathways such as toll-like receptors, cytokine receptors, or chemokine receptors (Giridharan and Srinivasan, 2018). Calcification in AFBs seems to be connected more to the diabetic environment and independent of RAGE signaling. More work needs to be performed to determine the role of TLR4 signaling in the calcification response in AFBs.

We also further explored oxidative stress responses by measuring H_2_O_2_ production and SOD-2 expression in VSMCs and AFBs. In VSMCs, H_2_O_2_ concentrations were significantly increased in diabetic cell types, which may correlate to a hyperglycemic environment (Patel et al., 2013; Tiwari et al., 2013). Patel et al. (2013) also described an increase in H_2_O_2_ production in a hyperglycemic environment in human endothelial cells. We also found that calcification and AGE treatment significantly increased H_2_O_2_ production in non-diabetic VSMCs, while non-diabetic RKO VSMCs remained the same. This finding was indicative of H_2_O_2_ production being linked to RAGE presence in non-diabetic VSMCs (Tiwari et al., 2013; Byon et al., 2016). In addition, AGE treatment of rat VSMC was demonstrated to increase ROS production levels (Wei et al., 2013). SOD-2 is a protein responsible for clearing mitochondrially-linked H_2_O_2_ (Zelko et al., 2002). In our study, diabetic VSMCs had significantly increased SOD-2 expression due to the application of AGEs without calcification treatment. Miller et al. (2008) found increased H_2_O_2_ production and decreased SOD-1, SOD-2, and SOD-3 expression and activity in calcified aortic valves. It was suggested enzyme recycling may lower SOD-2 expression in calcification groups. While diabetic RKO VSMCs had significantly higher levels of SOD-2 expression in all treatments, we believe the enzyme may either not be active or the hyperglycemic environment significantly increased SOD-2 expression (Vincent et al., 2007). Future studies will include determining if activity of SOD-2 has been diminished in VSMCs. Unlike VSMCs, all genotypes of AFBs had a significantly higher concentration of H_2_O_2_ due to calcification and AGE treatments, suggesting H_2_O_2_ production was not AGE/RAGE dependent in AFBs. Diabetic AFBs had the highest expression of SOD-2, which was expected and could link to higher levels of AGE-mediated signaling and hyperglycemia (Yao and Brownlee, 2010). Overall, VSMCs and AFBs had different concentrations of H_2_O_2_ production and SOD-2 expression patterns as a result of calcification and AGE treatments. Therefore, the ability of these two cell types to differentially respond to calcification and exogenous AGE treatments opens the possibility for ROS stimulation to drive the severity of VC in the microenvironment of aorta.

The authors would like to acknowledge that there are limitations with this study, such as only confirming the AFBs cell type with one phenotype marker, vimentin. Some literature has shown that this marker is not enough to fully confirm fibroblast phenotype, but physical separation is a commonly utilized practice in the field to avoid cross-contamination, and other manuscripts have confirmed the fibroblast phenotype with similar isolation methods presented here (Ross, 1971; Li et al., 2000, 2002; An et al., 2007; Bruijn et al., 2020). The authors would also like acknowledge the presence of only one osteogenic phenotype marker, OPN. We would have liked to have included additional markers, but due to the current experimental set-up, other markers did not work in our laboratory. Although this is a limitation, other manuscripts have shown the presence of additional markers with similar *in vitro* culture models to the one presented here (Tanikawa et al., 2009; Li et al., 2020). Finally, this manuscript only includes one ROS assay, H_2_O_2_, which is a limitation. In the future, experimental design should be modified to include other ROS assays during sample collection.

Understanding individual cellular signaling pathways in health and disease is vital for dissecting how cells participate in intercellular communication. This work sought to show VSMCs and AFBs follow different pathways when responding to calcification conditions and stimulation with exogenous AGEs. While these cell types are physically located in an actively remodeling microenvironment during VC *in vivo*, they respond to calcification and AGE-initiated stress very differently. In the future, work should address how the molecules released into the microenvironment of each cell type to alter the behavior and phenotype of the opposite cell type in a paracrine-like fashion to impact vascular calcification in the diabetic patient.

## Data Availability Statement

The datasets presented in this study can be found in online repositories. The names of the repository/repositories and accession number(s) can be found below: Figshare https://doi.org/10.6084/m9.figshare.c.5323241.

## Ethics Statement

The animal study was reviewed and approved by University of Mississippi Animal Care and Use Committee IACUC protocol number 20-017.

## Author Contributions

AK and JS contributed to experimental design and laboratory experiments, revised, read, and approved the submitted version. AK performed all statistical analysis and graphing and wrote the first draft of the manuscript. Both authors contributed to the article and approved the submitted version.

## Conflict of Interest

The authors declare that the research was conducted in the absence of any commercial or financial relationships that could be construed as a potential conflict of interest.

## Abbreviations

AGEs: advanced glycation end products
AFBs: adventitial fibroblasts
ERK: extracellular-related kinase
MAPK: mitogen-activated protein kinase
NF-B: nuclear factor-κB
OPN: Osteopontin
RKO: RAGE knockout
RAGE: receptor for advanced glycation end products
SOD-2: superoxide dismutase 2
TLR4: toll-like receptor 4
T2DM: type 2 diabetes mellitus
VC: vascular calcification
VSMC: vascular smooth muscle cells
α-SMA: α-smooth muscle actin.
