# Supplementary material for: RAGE Differentially Altered in vitro Responses in Vascular Smooth Muscle Cells and Adventitial Fibroblasts in Diabetes-Induced Vascular Calcification
Source: Front Physiol. 2021 Jun 7;12:676727. doi: 10.3389/fphys.2021.676727 (PMC8215351; doi:10.3389/fphys.2021.676727)
Supplement: Supplementary file 1 [file Data_Sheet_1.PDF]

Supplementary Material

1 Supplementary Table 1

Supplementary Table 1: Animal Information					
	**Non-diabetic (n=124)**	**Non-diabetic RKO (n=94)**	**Diabetic (n=69)**	**Diabetic RKO (n=29)**	**P Value**
**Body Weight (g)**	29.34 ± 0.3051	31.68 ± 0.3041	51.34 ± 0.6148	56.91 ± 0.8943	P<0.0001
**Blood Glucose (mg/dL)**	194.3 ± 3.652	216.2 ± 3.906	529.3 ± 12.84	421.9 ± 22.38	P<0.0001

P Value derived from one-way ANOVA between all genotypes.

2 Supplementary Figures

2.1 Supplementary Figure 1

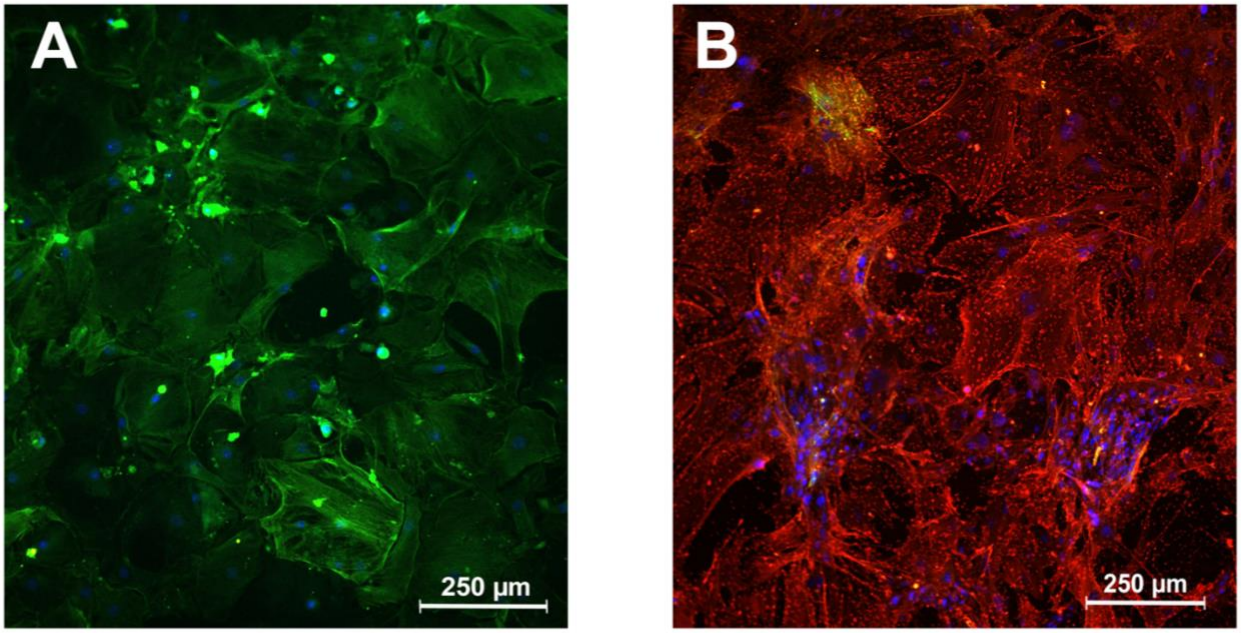

Supplementary Figure 1. Immunohistochemistry staining demonstrates purity of aortic cell isolations. Cells were fixed in 4% paraformaldehyde and imaged at 10X. VSMCs (**A**) were imaged with the following: α-SMA (green) and DAPI (blue). AFBs (B) were imaged with the following: α-SMA (green), vimentin (red), and DAPI (blue).

2.2 Supplementary Figure 2

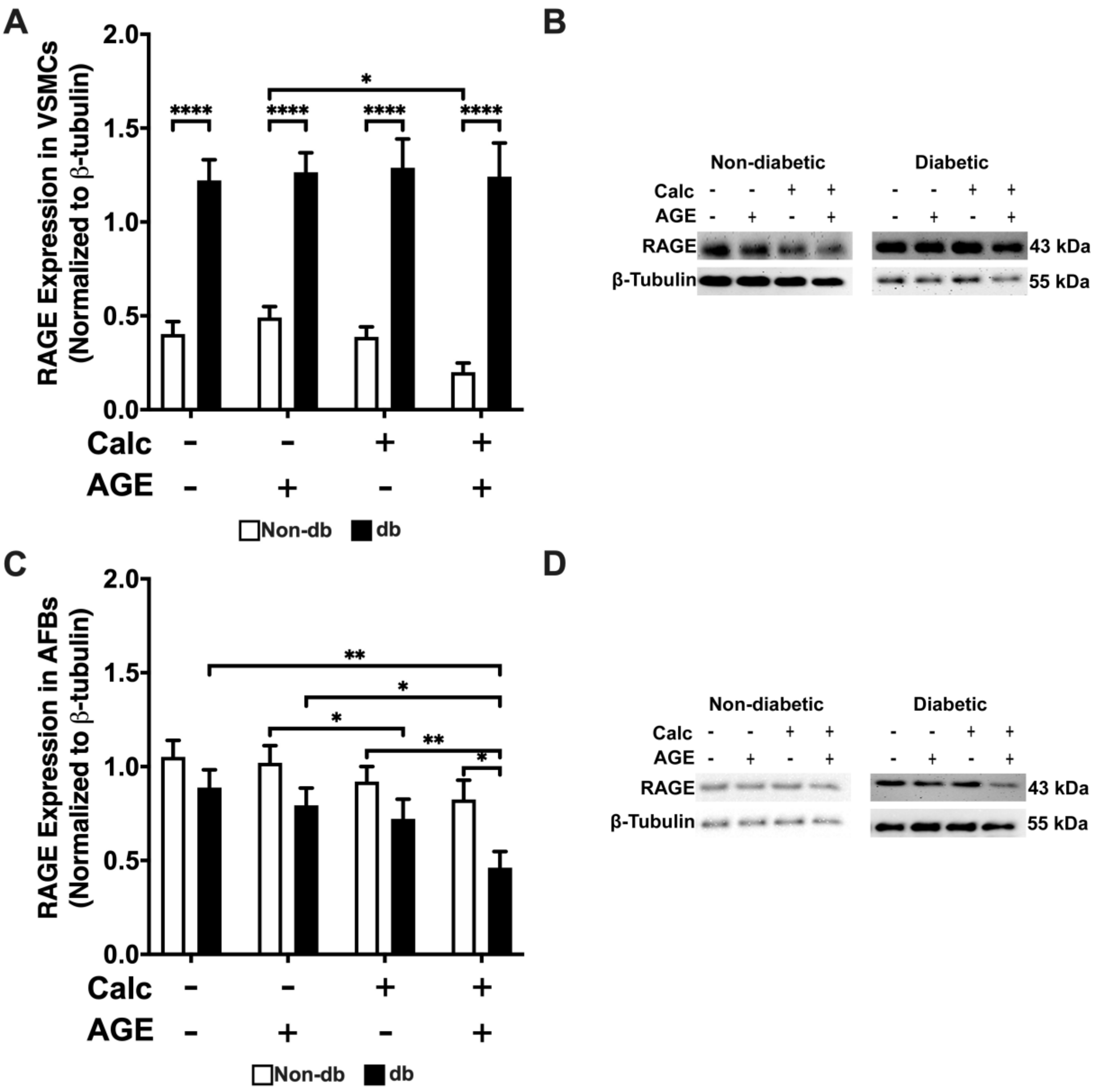

Supplementary Figure 2. Diabetic VSMCs express significantly more RAGE than non-diabetic VSMCs, while diabetic AFBs lose RAGE expression with calcification treatment. Protein expression of RAGE (43 kDa, **A & C)** was quantified. Non-diabetic, non-diabetic RKO, diabetic, and diabetic RKO primary VSMCs and AFBs were isolated and treated with 3 mM Pi with or without AGEs (0.5 mg/mL) for 7 days. Protein levels were normalized to β-tubulin (**B&D**) and graphed as mean ± SEM with n = 6-9 of independent replicates. Statistical analysis consisted of a one-way ANOVA and a Protected Fisher's LSD test post-hoc (* $p<0.05$, ** $p<0.01$, *** $p<0.001$, **** $p<0.0001$).
